# Monitored data on occupants’ presence and actions in an office building

**DOI:** 10.1038/s41597-019-0271-7

**Published:** 2019-11-26

**Authors:** Ardeshir Mahdavi, Christiane Berger, Farhang Tahmasebi, Matthias Schuss

**Affiliations:** 1 Department of Building Physics and Building Ecology, TU Wien, Vienna, Austria; 2 Institute for Environmental Design and Engineering, University College London, London, UK

**Keywords:** Civil engineering, Energy modelling

## Abstract

Within a study, an open plan area and one closed office in a university building with a floor area of around 200 m^2^ were monitored. The present data set covers a period of one year (from 2013-01-01 to 2013-12-31). The collected data pertains to indoor environmental conditions (temperature, humidity) as well as plug loads and external factors (temperature, humidity, wind speed, and global irradiance) along with occupants’ presence and operation of windows and lights. The monitored data can be used for multiple purposes, including the development and validation of occupancy-related models.

## Abstract (summary)

**Table:** 

Measurement(s)	Occupancy • room temperature ambient air • humidity • radiation • temperature of air • atmospheric wind speed • atmospheric wind direction • electrical energy
Technology Type(s)	sensor • Gauge or Meter Device
Sample Characteristic - Organism	Homo sapiens
Sample Characteristic - Environment	office building
Sample Characteristic - Location	Vienna

Machine-accessible metadata file describing the reported data: 10.6084/m9.figshare.9822623

## Background & Summary

Professionals in the building design, construction, and operation have become increasingly aware concerning the importance and value of monitored data from buildings^1^. Such data can support the objective assessment of buildings’ indoor environmental conditions and energy performance. As such, building delivery and commissioning process cannot be considered accountable without an evidence-based monitoring-supported verification^2^. Moreover, monitored data can support operational optimisation of existing building stock and – accumulated over time and multiple buildings – inform and improve future projects. Energy and performance contracting, smart load balancing, model-predictive building systems control, and preventive building maintenance can all significantly benefit from systematic collection and analysis of monitored data. Likewise, high-quality data can contribute to the state of knowledge in areas such as building physics, building integrity, building automation, indoor environment, and human factors in building performance.

The data included in the present contribution represents a part of an effort toward systematic and comprehensive data collection in an existing office building. The associated process facilitated a better understanding of the shortcomings in the current practices concerning the technical infrastructures for building monitoring and related challenges in hardware scalability and software interoperability. Moreover, the multi-aspect nature of the collected data support the process of ontology development for building-related monitored data. This ontology may be described in terms of a general schema or a structured matrix of multiple data streams originating from, and relevant to, the operation of buildings. This ontology^2,3^ has been shown to have the potential to enhance the understanding of building-related data space and provide a solid foundation for further developments with respect to applications in building data acquisition, storage, processing, and analysis.

## Methods

**Fig. 1:**
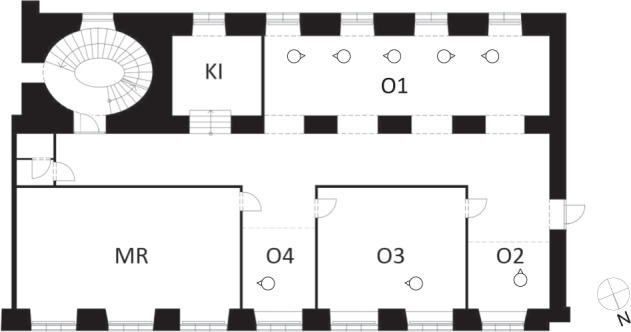
Floor plan office area. The abbreviation KI stands for kitchen, O1 for office 1, O2 for office 2, O3 for office 3, O4 for office 4, and MR for meeting room.

The present contribution represents the data monitored over a period of one year (from 2013- 01-01 to 2013-12-31) in an office area of around 200 m^2^ in a university building in Vienna. Figure 1 illustrates the setting and monitoring infrastructure of the office area. Within this study, multi-aspect (thermal, visual, and equipment) data of this office area as well as external conditions are collected.

Table 1 shows the measured variables within this study including information about inhabitants (around eight occupants were monitored), indoor and external conditions, control systems/devices and equipment.

**Table 1:** Measured variables.

Categories of measured data	Subcategories of measured data	Specific variables you measured
Inhabitants	Position	Presence at work station
	Control action	Light/Window
Indoor conditions	Hygro-thermal	Air temperature/Air relative humidity
External conditions	Hygro-thermal	Air temperature/Air relative humidity/Wind speed/Wind direction
	Solar radiation	Global radiation
Control systems/devices	Lighting	On/off
Equipment	Office	Equipment power

The area layout (see Fig. 1) includes a single-occupancy closed office (O3), two single- occupancy semi-closed offices (O2, O4), an open plan office area (O1), a kitchen (KI), and a meeting room (MR).

**Fig. 2:**
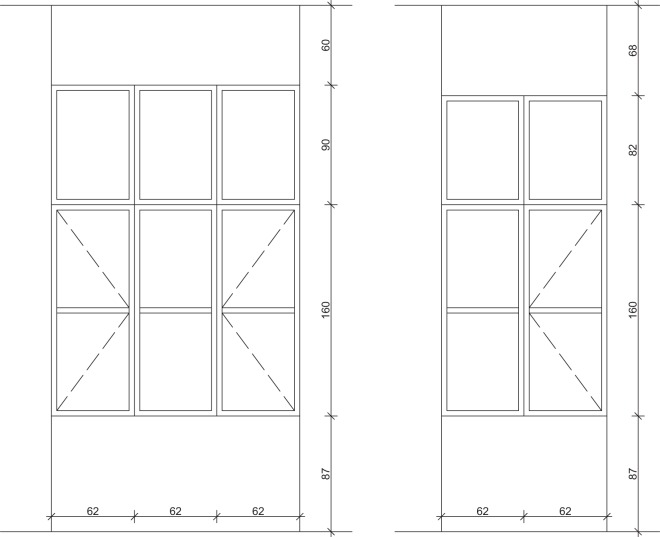
Casement window dimensions. The dimensions of south-facing windows are given on the left, the dimensions of north-facing windows are given on the right.

The naturally ventilated office area includes eight workstations, in which each occupant has access to one manually operable casement window. Only the enclosed office entails one workstation and two windows. Opening and closing actions are typically conducted on operable internal and external wings of the casement windows. Each window is equipped with internal shading elements. Dimensions of the casement window are given in Fig. 2. Occupants’ window opening behaviour is not likely to have been influenced by circumstances such as traffic noise or poor air quality, given low external ambient sound levels (windows are oriented toward internal courtyards) and relatively low (measured) CO_2_ levels.

The occupants’ presence, state of windows and a number of indoor environment variables (including indoor air temperature, indoor air relative humidity, and equipment power) are monitored on a continuous basis. The arrangement of the monitoring infrastructure within the office area is given in Table 2.

**Table 2:** Monitored variables at each office.

Office	Floor area [m^2^]	Presence at work station	Window control actions	Lighting on/off	Equipment power	Indoor air temp.	Indoor air relative humidity
KI	11	Pki	Wki	Lki		Tki	Rhuki
O1	36	Po1_1	Wo1_1	Lo1_1	Eo1_1	To1_1	Rhuo1_1
		Po1_2	Wo1_2	Lo1_2	Eo1_2	To1_2	Rhuo1_2
		Po1_3	Wo1_3		Eo1_3		
		Po1_4	Wo1_4		Eo1_4		
		Po1_5			Eo1_5		
O2	11	Po2	Wo2_1	Lo2	Eo2	To2	Rhuo2
			Wo2_2				
O3	29	Po3	Wo3_1	Lo3_1	Eo3	To3	Rhuo3
			Wo3_2	Lo3_2			
			Wo3_3				
			Wo3_4				
O4	11	Po4	Wo4_1	Lo4_1	Eo4	To4	Rhuo4
			Wo4_2	Lo4_2			
MR	44		Wmr_1			Tmr	Rhumr
			Wmr_2				
			Wmr_3				
			Wmr_4				
			Wmr_5				
			Wmr_6				

Table 3 provides an overview of the monitoring infrastructure. Data collection of the indoor climate and the user interactions within the office area was performed with an in-house developed monitoring system concept based on off-the-shelf wireless EnOcean sensors, a wireless telegram data collector and a central web-based monitoring service^4^.

**Table 3:** Elements of the monitoring infrastructure.

Sensor type	Measured variable	Range	Accuracy
Thermokon - SR04 CO2 rH	Indoor air temperature	0–51 °C	±1% of measuring range (typ. at 21 °C)
	Indoor air relative humidity	0–100%rH	±3% between 20–80% rH (typ. at 21 °C)
	CO_2_	0–2550 ppm	±75 ppm or ±10% of measuring range (typ.at 21 °C)
Thermokon - SR-MDS Solar	Motion/occupancy	0/1	—
	Brightness	0–512Lux	—
Thermokon - SRW01	Window contact sensor	0/1	—
Eltako - FWZ61	Single phase energy meter	0–3680 W	±1%
Thies Clima - Pyranometer CM3	Solar radiation	0–1300 W.m^−2^	±5% (350–1500 nm)
Thies Clima – Hygro-Thermogeber-compact 1.1005.54.000	Air temperature	−30–+70 °C	±0.2 K at 20 °C and wind speed >1.0 m.s^−1^
	Air relative humidity	0–100% rH	±2% rH
Thies Clima - Windgeber-compact 4.3519.00.000	Wind speed	0.5–50 m.s^−1^	±0.5 m/s or ±3% of measurement
Thies Clima - Windrichtungsgeber- compact 4.3129.00.000	Wind direction	0–360°	±5°

In detail, the occupancy data has been obtained via wireless ceiling-mounted PIR motion detectors. The sensors are active in interval of 1.6 minutes and detect movements and measure the brightness at ceiling level. Like all sensors based on EnOcean standard, the transmission of the telegrams is reduced to a necessary minimum. The resulting low energy demand is usually covered by a solar cell or a battery. As a result, telegrams were only sent when a value change is higher than a sensor specific minimum or a maximum time difference to the previous telegram was exceeded. The recorded data entails a sequence of time- stamped occupant motion detection with binary values. In order to facilitate data analysis, the event-based data streams were processed to generate 15-minute interval data by the use of stored procedures implemented in the MySQL database of the MOST building monitoring system^5,6^. In case of occupancy, this stored procedure derives the duration of occupancy states (occupied/vacant) from the stored events and returns the dominant occupancy state of each interval.

Indoor air temperature and relative humidity were measured inside each office area close to each workstation at 0.9 m height. The state of all windows was measured through a window contact sensor. Light state was indirectly measured by the use of an EnOcean-based electric energy meter. The recorded event-based data from the EnOcean-based sensors was subsequently processed by a stored procedure of the MOST building monitoring system to generate the values of the provided data records for each time interval^6^.

Outdoor environmental parameters (including air temperature, air relative humidity, wind speed, wind direction, and global radiation) are monitored via a local weather station.

The weather station is located on top of the building at about 40 m above street level. No obstacles were situated close by that could potentially influence the wind direction or speed.

## Data Records

Table 4 provides an overview of the data records. Data file names, format types as well as measured variables are described. The data records^7^ are stored in CSV format on the figshare repository. Key to the location codes is provided in Fig. 1.

**Table 4:** Data records.

Data file name	Data format	Location code	Measured variable
01_occ	csv	O1, O2, O3, O4, KI	Presence at work station [0:vacant, 1:occupied]
02_win	csv	O1, O2, O3, O4, MR, KI	Window state [0:open, 1:closed]
03_light	csv	O1, O2, O3, O4, KI	Light state [0:off, 1:on]
04_plug	csv	O1, O2, O3, O4	Equipment power [W]
05_temp_in	csv	O1, O2, O3, O4, MR, KI	Indoor air temperature [°C]
06_rhu_in	csv	O1, O2, O3, O4, MR, KI	Indoor air relative humidity [%]
07_rad_global	csv	Weather station	Global radiation [W.m^−2^]
08_temp_out	csv	Weather station	Air temperature [°C]
09_rhu_out	csv	Weather station	Air relative humidity [%]
10_wsp	csv	Weather station	Wind speed [m.s^−1^]
11_wdi	csv	Weather station	Wind direction [degree] (North:0, East:90, South:180, West:270)

## Technical Validation

The data included in the present contribution displays an inherently multi-layered nature, involving multiple domains (thermal, visual, equipment) and multiple probes (of different type and built). Moreover, it is relayed and stored via multiple technologies, and processed to fit different categories within the underlying monitoring ontology. Given this nature, evidence of technical validation cannot be presented in terms of a single experimental design. Nonetheless, the long-term data collection and processing effort incorporated a number of measures and operations to ensure the consistency and reliability of the data set. These include:

(i) Regular calibration of sensory probes: This was conducted both by third-party instances every three years (specifically regarding the weather station sensors) and via Department’s own climate-chamber for temperature probe output comparison before installation and thereafter (via annual comparisons with a reference probe);
(ii) Systemic comparison of output of the probes of the same kind when placed in the same positions;
(iii) Recurrent standard quality and plausibility checks toward preventive detection of potential probe output disruption, malfunction, or corruption;
(iv) Post-repository pre-submission data distillation excluding all but those data elements meeting the above criteria.
